# Structure of a photosynthetic reaction centre determined by serial femtosecond crystallography

**DOI:** 10.1038/ncomms3911

**Published:** 2013-12-19

**Authors:** Linda C. Johansson, David Arnlund, Gergely Katona, Thomas A. White, Anton Barty, Daniel P. DePonte, Robert L. Shoeman, Cecilia Wickstrand, Amit Sharma, Garth J. Williams, Andrew Aquila, Michael J. Bogan, Carl Caleman, Jan Davidsson, R Bruce Doak, Matthias Frank, Raimund Fromme, Lorenzo Galli, Ingo Grotjohann, Mark S. Hunter, Stephan Kassemeyer, Richard A. Kirian, Christopher Kupitz, Mengning Liang, Lukas Lomb, Erik Malmerberg, Andrew V. Martin, Marc Messerschmidt, Karol Nass, Lars Redecke, M Marvin Seibert, Jennie Sjöhamn, Jan Steinbrener, Francesco Stellato, Dingjie Wang, Weixaio Y. Wahlgren, Uwe Weierstall, Sebastian Westenhoff, Nadia A. Zatsepin, Sébastien Boutet, John C.H. Spence, Ilme Schlichting, Henry N. Chapman, Petra Fromme, Richard Neutze

**Affiliations:** 1 Department of Chemistry and Molecular Biology, University of Gothenburg, 405 30 Gothenburg, Sweden; 2 Center for Free-Electron Laser Science, DESY, Notkestrasse 85, 22607 Hamburg, Germany; 3 Max-Planck-Institut für Medizinische Forschung, Jahnstrasse 29, 69120 Heidelberg, Germany; 4 Max Planck Advanced Study Group, Center for Free-Electron Laser Science, Notkestrasse 85, 22607 Hamburg, Germany; 5 Linac Coherent Light Source, LCLS, SLAC National Accelerator Laboratory, 2575 Sand Hill Road, Menlo Park, 94025 California, USA; 6 PULSE Institute, SLAC National Accelerator Laboratory, 2575 Sand Hill Road, Menlo Park, 94025 California, USA; 7 Department of Chemistry-Ångström Laboratory, Uppsala University, Box 523, 75120 Uppsala, Sweden; 8 Department of Physics, Arizona State University, Tempe, 85287 Arizona, USA; 9 Lawrence Livermore National Laboratory, 7000 East Avenue, Livermore, 94550 California, USA; 10 Department of Chemistry and Biochemistry, Arizona State University, Tempe, 85287-1604 Arizona, USA; 11 Department of Physics, University of Hamburg, Luruper Chaussee 149, 22761 Hamburg, Germany; 12 Joint Laboratory for Structural Biology of Infection and Inflammation, Institute of Biochemistry and Molecular Biology, University of Hamburg and Institute of Biochemistry, University of Lübeck at DESY, 22607 Hamburg, Germany

## Abstract

Serial femtosecond crystallography is an X-ray free-electron-laser-based method with considerable potential to have an impact on challenging problems in structural biology. Here we present X-ray diffraction data recorded from microcrystals of the *Blastochloris viridis* photosynthetic reaction centre to 2.8 Å resolution and determine its serial femtosecond crystallography structure to 3.5 Å resolution. Although every microcrystal is exposed to a dose of 33 MGy, no signs of X-ray-induced radiation damage are visible in this integral membrane protein structure.

## Abstract (web summary)

Serial femtosecond crystallography is an X-ray free-electron-laser-based method that uses X-ray bursts to determine protein structures. Here the authors present the structure of a photosynthetic reaction centre, an integral membrane protein, achieved with no sign of X-ray-induced radiation damage.

## Graphical abstract

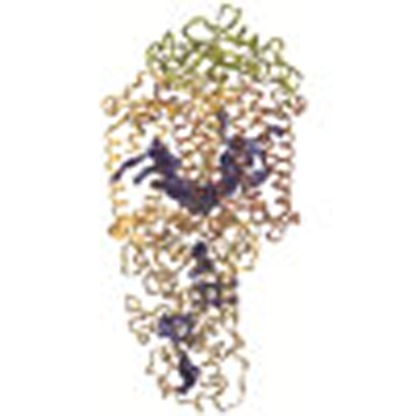

X-ray crystallography is the most successful method for determining new membrane protein structures. Membrane protein crystallization, however, continues to pose many challenges because of the inherent conflict between hydrophilic and hydrophobic domains within the protein, flexible loops and low protein expression yields^1^. When crystals are obtained they are often small, frequently diffract to low resolution, and crystal collection and cryoprotection can introduce additional challenges. As such, it has become increasingly common to use synchrotron-based microfocus beamlines^2^ to screen large numbers of very small membrane protein crystals and to merge diffraction data from multiple crystals so as to recover complete diffraction data.

Serial femtosecond crystallography (SFX) brings a new dimension to the challenge of screening small crystals. This approach has been developed as a high-resolution structural method^3^,^4^,^5^,^6^ at X-ray free-electron lasers (XFELs) and allows diffraction data to be collected at room temperature from thousands of randomly oriented, fully hydrated micron-sized crystals injected into the XFEL beam using a microjet^7^,^8^. In combination with rapid X-ray detectors^9^, it is possible to collect millions of frames in a 12 hour shift at the Linac Coherent Light Source (LCLS)^10^, which provides extremely intense X-ray pulses at a repetition rate of 120 Hz. In addition to microfocusing, the key advantage of XFEL-based serial femtosecond crystallography over conventional diffraction strategies using synchrotron radiation is that each XFEL pulse of ∼10^12^ X-ray photons is only a few tens of femtoseconds in duration. This allows diffraction data to be recorded using brilliant X-ray bursts before the microcrystals have time to be destroyed by the effects of radiation damage^11^,^12^,^13^.

The first proof-of-principle SFX structures were of photosynthetic membrane protein complexes^3^,^4^ up to 8.2 Å resolution and were recovered from diffraction data recorded at the atomic, molecular and optical science experimental station^14^ of the LCLS^10^ using the CAMP instrument^15^. High-resolution SFX structures of the soluble proteins lysozyme^5^ and cathepsin B^6^ (1.9 and 2.1 Å resolution, respectively) were reported following the commissioning of the coherent X-ray imaging (CXI) instrument^16^, which operates at shorter wavelengths. Despite these advances, diffraction data recorded at the LCLS from membrane protein microcrystals have been considerably weaker than data recorded from microcrystals of soluble proteins. The highest-resolution membrane protein SFX structure reported to date is from photosystem II (ref. 17) and extends only to 5.7 Å resolution, a resolution at which side chains cannot usually be resolved.

In this work, microcrystals of the photosynthetic reaction centre from *Blastochloris viridis* (RC_*vir*_), a four-subunit integral membrane protein complex of 135 kDa, are grown in a lipidic sponge phase (LSP)^4^ and exposed to the focused XFEL beam of the LCLS^10^. Diffraction data from 1,175 crystals are merged to recover a SFX structure to 3.5 Å resolution. Although the X-ray exposure for each and every microcrystal is 33 MGy, which is approximately two orders of magnitude beyond that normally suitable for room temperature data collection, no signs of radiation damage are visible in the resulting electron density. These results demonstrate the considerable potential of XFEL-based SFX when applied to the challenging field of membrane protein structural biology.

## Results

### Diffraction data and electron density maps

**Figure 1:**
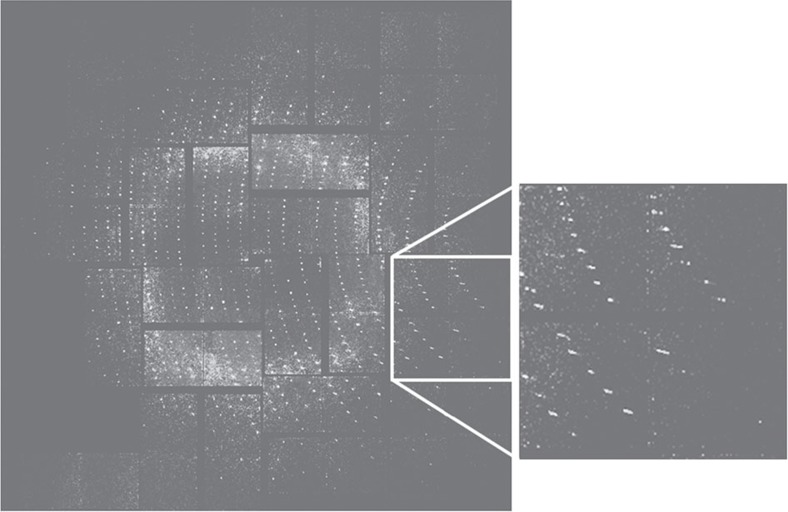
SFX diffraction data. Data were recorded on the Cornell-SLAC Pixel Array detector from microcrystals of RC_*vir*_ at the CXI beamline of the LCLS. The resolution limit at the edge of the detector was 2.62 Å. Diffractions spots were observed up to 2.8 Å resolution.

Microcrystals of RC_*vir*_ were grown in a LSP^4^ and transported at room temperature to the LCLS. LSP crystallization^18^,^19^ is a liquid analogue of the lipidic cubic phase crystallization^19^,^20^,^21^, which aids membrane protein crystal growth by forming a continuous lipidic bilayer mimicking the natural membrane environment. The LSP-grown RC_*vir*_ microcrystals were injected as a microjet^7^,^8^ across the focused XFEL beam of the LCLS and diffraction data to 2.8 Å resolution (Fig. 1) were collected on a Cornell-SLAC Pixel Array detector^9^ at the CXI instrument^16^. As this beamline operates at a short wavelength (∼1.4 Å), it was possible to extend our earlier 8.2 Å resolution RC_*vir*_ SFX structure^4^ to 3.5 Å resolution, at which most side chains can be assigned without ambiguity.

From two million images recorded during 5 h of beamtime, 3% were identified as containing more than ten spots using the software Cheetah^22^, of which 5,767 showed diffraction when examined visually. Indexing was performed using the CrystFEL suite^23^ and resulted in 1,175 processed diffraction images, which were scaled and merged using Monte Carlo methods^24^. Diffraction data were cut at 3.5 Å resolution for which *I*/*σ*≥2 and the completeness ≥92% (Supplementary Fig. S1, Table 1 and Supplementary Table S1). The RC_*vir*_ structure was solved by molecular replacement, yielding the same crystal packing (space group P2_1_2_1_2_1_; *a*=57.9 Å, *b*=84.8 Å, *c*=384.3 Å) as previously reported^4^. This is compared with the crystal packing of other RC_*vir*_ structures in Supplementary Table S2.

**Figure 2:**
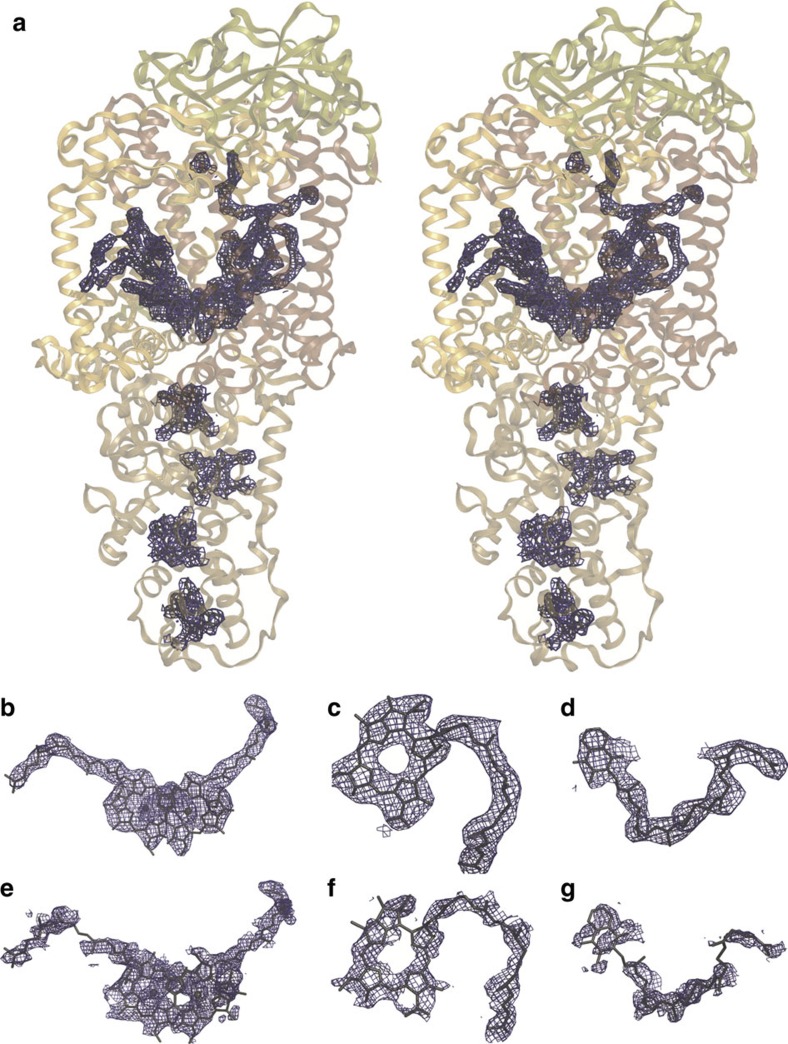
**Electron density maps calculated from the SFX RC**_***vir***_ **diffraction data.** (**a**) Stereo view of the 2mF_obs_−DF_calc_ electron density map for the RC_*vir*_ cofactors. Close-up views of the 2mF_obs_−DF_calc_ electron density map are shown for (**b**) the special pair, (**c**) the L-branch bacteriopheophytin and (**d**) the menaquinone in the *Q*_A_ pocket. Close-up views of the composite omit electron density map are shown for (**e**) the special pair, (**f**) the L-branch bacteriopheophytin and (**g**) the menaquinone. The composite omit map, which suffers from less model bias than the 2mF_obs_−DF_calc_ electron density map, better illustrates the quality of the SFX diffraction data. All panels are contoured at 1.0*σ*.

Figure 2a shows the 2mF_obs_−DF_calc_ electron density map in stereo contoured around all cofactors after structural refinement to 3.5 Å resolution, for which *R*-factor and *R*_free_ values of 29.4 and 32.7% were recovered. A closer view of this electron density illustrates that the bacteriochlorophyll tails of the special pair are unbroken (Fig. 2b); that the bacteriopheophytin cofactors do not have a metal centre (Fig. 2c); and that the menaquinone head group and tail are resolved within the *Q*_A_ pocket (Fig. 2d). Similarly, the electron density recovered when calculating the composite omit map (Fig. 2e–g), which displays less model bias than the 2mF_obs_−DF_calc_ map, also reveals clear electron density for these cofactors. As key quality indicators such as *I*/*σ*, CC_1/2_, completeness and *R*_free_ vary smoothly as the resolution is extended (Supplementary Fig. S1), our choice of cutting the data to 3.5 Å resolution might be viewed as unnecessarily conservative. Calculation of the composite omit map at 3.3 Å resolution (Supplementary Fig. S2), however, does not reveal new details within the map and the electron density for the cofactors appears more broken than when the data are cut at 3.5 Å resolution.

The quality of the electron density maps at 3.5 Å resolution is striking given that the overall multiplicity is 27 (Table 1) and is orders of magnitude lower than that reported for high-resolution SFX structures of lysozyme^5^ (379) and cathepsin B^6^ (7,808); there is considerable variation in the crystallographic observations that are averaged to recover *I*_hkl_ for each and every hkl value^24^ (Supplementary Fig. S3 illustrates sets of observations for representative hkl values); and data processing with a long axis (*c*=384.3 Å) introduces experimental challenges due to the close spacing of diffraction spots in reciprocal space. As a result, the correlation coefficient of the SFX data when split into two halves is lower when projected along the *c* axis relative to that recovered when projecting along the *a* or *b* axes (Supplementary Fig. S1a,b). These findings indicate the power of SFX multicrystal averaging to recover high-multiplicity data sets for which the electron density maps are better than normally expected using traditional quality indicators developed for single crystal data sets.

### Radiation damage

A groundbreaking aspect of XFEL-based SFX is that the use of ultrafast X-ray pulses enables the traditional radiation damage barrier of structural biology^25^ to be side-stepped^11^,^12^. In this work, each microcrystal was exposed to a 42-fs X-ray pulse of 4 × 10^11^ 9.34 keV photons focused into a spot of 10 μm^2^. This corresponds to a dose of 33 MGy deposited into each and every microcrystal (calculated using RADDOSE^26^), which equates to instantaneously depositing the heat output of a 1-kW kettle running for 9 h into 1 litre of water (1 MGy=1 MJ absorbed by 1 kg of matter). This dose is approximately two orders of magnitude above that normally acceptable for traditional room-temperature crystallography and is equal to an empirical X-ray dose limit found at cryogenic temperatures^25^. Under these conditions, approximately four primary photo-ionization events occur within every RC_*vir*_ molecule, which in turn induce a further 200 secondary ionization events^27^.

**Figure 3:**
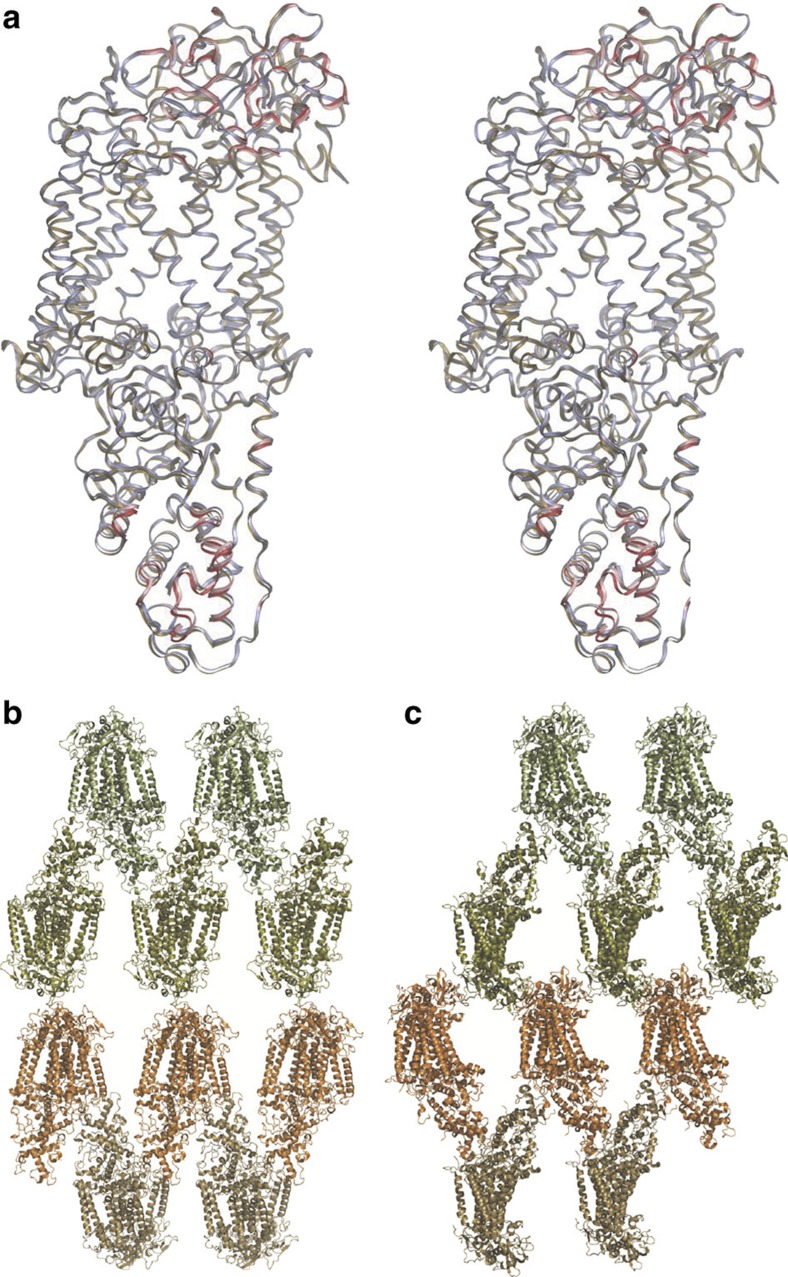
**Comparison of the RC**_***vir***_ **SFX structure with an earlier structure.** (**a**) Superposition in stereo of the SFX crystal structure (brown) upon the Laue diffraction RC_*vir*_ structure (white, pdb entry 2X5U). Both structures were aligned on their 11 TM helices. Regions for which the coordinate changes are ≥1.0*σ* (calculated according to ref. 29, Supplementary Fig. S4) are marked in red and correspond to subtle backbone differences in subunits C and H. (**b**) Crystal packing of the LSP microcrystal form used in this SFX study. (**c**) Crystal packing of the LSP macrocrystal form (that is, larger crystals) used in other studies^28^,^30^. Crystal contacts differ between the microcrystal (space group P2_1_2_1_2_1_; *a*=57.9 Å; *b*=84.8 Å; *c*=384.3 Å) and macrocrystal (space group P2_1_2_1_2; *a*=85 Å; *b*=139 Å; *c*=178 Å) forms.

As with studies of lysozyme under similar experimental conditions^5^, the RC_*vir*_ electron density appears unaffected by these damaging events. First and foremost, the SFX crystal structure of RC_*vir*_ is very similar to an earlier room-temperature Laue diffraction structure^28^ (pdb entry 2X5U) having a root mean squared deviation (r.m.s.d.) of 0.49 Å calculated over all *C*_α_ atoms when both RC_*vir*_ models are aligned on the 11 transmembrane (TM) α-helices. All *C*_α_ changes have a statistical significance ≤2.0*σ* and the largest changes (error-weighted difference ≥1.0*σ* relative to at least 20 other *C*_α_ atoms) are associated with the C- and H-subunits (Fig. 3a), as illustrated by calculation of the error-weighted change in internal distance matrix^29^ (Supplementary Fig. S4). These differences correspond to an r.m.s.d. shift of 0.71 Å for the C subunit and 0.48 Å for the H-subunit when aligned on the RC_*vir*_ TM regions (Supplementary Table S3). Although the crystal packing of both microcrystal and macrocrystal (that is, normal-sized crystals typically 0.2 × 0.2 × 0.5 mm^3^) forms are similar, with both forming stacked layers of two-dimensional membrane crystals, there are differences between the protein:protein crystal packing interactions that are largely mediated by the C- and H-subunits (Fig. 3b,c), and these presumably underlie this structural perturbation.

**Figure 4:**
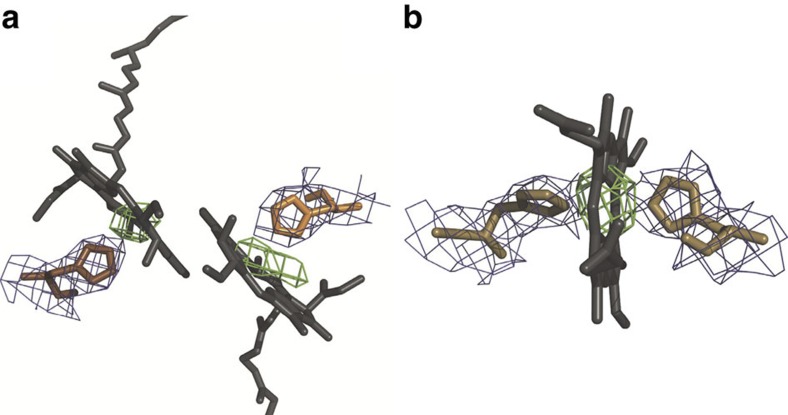
Electron density for the metal containing cofactors. (**a**) mF_obs_−DF_calc_ residual electron density (green, contoured at 1.5*σ*) calculated with the two Mg atoms removed from the special pair. (**b**) mF_obs_−DF_calc_ residual electron density (green, contoured at 3.0*σ*) calculated with the Fe atom removed from haem 4. The 2mF_obs_−DF_calc_ electron density map (blue, contoured at 1.0*σ*) shows the electron density for the histidine side chains coordinating (**a**) the Mg atoms and (**b**) the iron.

Calculation of the mF_obs_−DF_calc_ omit electron density map (Fig. 4) with all Fe and Mg atoms removed from the model shows that all metal centres, which have higher photo-ionization cross-sections than other atoms in the sample, emerge as strong positive residual density peaks. The average significance of the residual mF_obs_−DF_calc_ omit map peaks for the Mg atoms was 3.0*σ*, whereas that for the Fe atoms was 6.0*σ*, corresponding to a ratio for Mg:Fe of 50% and in agreement with that recovered (48%) using synchrotron diffraction data to 1.9 Å resolution^30^ (pdb entry 2WJN). Furthermore, the 2mF_obs_−DF_calc_ electron density for the residues that ligate the bacteriochlorophyll and haem metal centres is well ordered (Fig. 4).

**Figure 5:**
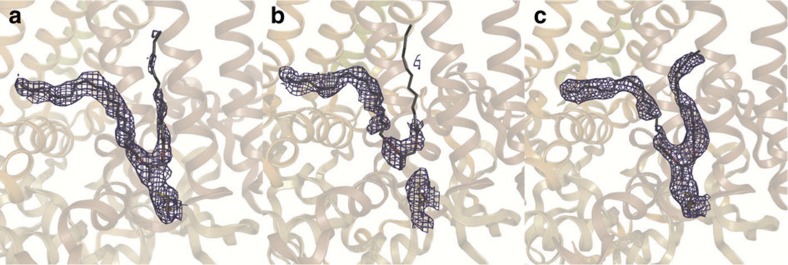
X-ray damage to a radiation-sensitive bond. (**a**) Electron density illustrating the thioether covalent bond linking the N-terminal cysteine of the C subunit to a diacylglycerol molecule calculated from monochromatic diffraction data^30^ collected at cryogenic temperatures using an X-ray dose of 4.4 MGy. (**b**) Electron density calculated when the X-ray dose at cryogenic temperatures was 77 MGy. (**c**) Electron density calculated from the room-temperature SFX diffraction data using an X-ray dose of 33 MGy. The continuous electron density in **a** is highly disrupted in **b**, illustrating how radiation damage cleaves this covalent thioether bond. In the room temperature SFX structure **c**, this bond appears unaffected by radiation damage. All panels are contoured at 0.7*σ*.

Radiation damage studies of RC_*vir*_ at cryogenic temperatures have identified that the covalent thioether bond linking the N-terminal cysteine residue of the tetrahaem cytochrome *c* subunit (CysC1) to a diacylglycerol molecule is susceptible to X-ray-induced damage. Specifically, a comparison of the electron density recovered from monochromatic data collected at 100 K using an X-ray dose of 4.4 MGy (Fig. 5a, pdb entry 2WJM^30^) with that recovered using a dose of 77 MGy at 100 K (Fig. 5b, pdb entry 2WJN^30^) reveals that this bond is completely cleaved at high dose. As the RC_*vir*_ SFX diffraction data were collected at room temperature, it might be anticipated that these data would be more prone to radiation-induced damage than data collected at cryogenic temperatures. Nevertheless, the 2mF_obs_−DF_calc_ SFX electron density map, calculated using a dose of 33 MGy per microcrystal, reveals continuous electron density for this bond (Fig. 5c). This finding clearly demonstrates that according to this internal radiation damage marker, there are no observable effects of radiation damage on the SFX crystal structure of RC_*vir*_.

## Discussion

XFELs, by providing extremely brilliant X-ray pulses of femtoseconds in duration, have the potential to have major impact on the field of structural biology. Earlier proof-of-principle studies demonstrated that SFX is a high-resolution method when studying microcrystals of soluble proteins^5^,^6^. From a long-term perspective, the impact of XFEL-based SFX on structural biology will depend on its ability to address problems that are challenging or intractable using traditional data collection strategies at synchrotron radiation sources. Membrane protein crystallography remains a major challenge for the structural biology community^1^. Our results establish that SFX structures can be recovered from LSP membrane protein microcrystals to a resolution where protein side chains, cofactors and bound lipid molecules can be unambiguously resolved. Moreover, no indications of radiation-induced damage are visible in radiation-sensitive covalent bonds or metal cofactors. These findings provide a platform from which future challenges in membrane protein structural biology can be addressed with confidence.

## Methods

### Purification and LSP batch crystallization

The photosynthetic reaction centre from *Bl. viridis* was purified^4^ using 250 ml POROS ‘50 micron’ HQ media (Applied Biosystems Europe BV) packed in an XK 50/20 column (GE Healthcare) equilibrated with 20 mM Tris-HCl, pH 8.5, and 1% lauryldimethylamine-oxide (LDAO). The protein was eluted using a linear gradient of 0–0.5 M NaCl in 20 mM Tris-HCl, pH 8.5, and 1% LDAO, and subsequently loaded onto a HiPrep 26/60 Sephacryl S-300 column (GE Healthcare) equilibrated with 10 mM Tris-HCl, pH 8.5, and 0.1% LDAO. This protocol yielded ∼3 mg of pure RC_*vir*_ per litre of cell culture. Batch crystallization trials were prepared in septum-sealed glass vials (Sigma-Aldrich) containing 100 μl protein (25–35 mg ml^−1^), 100 μl LSP (12% monoolein, 17.5% Jeffamine M-600, 1.0 M Hepes (pH 8.0), 0.7 M (NH_4_)_2_SO_4_ and 2.5% 1,2,3-heptanetriol) and 100 μl of 1.0–1.2 M trisodium citrate. The setups were left to equilibrate for 2–4 weeks at 20 °C and were shipped at room temperature to the LCLS.

### Sample injection and data collection

RC_*vir*_ crystals were delivered to the injector nozzle via a syringe^31^ and were injected into the XFEL beam at a flow rate of 10 μl min^−1^. The liquid capillary of the nozzle had an inner diameter of 100 μm and the liquid was focused by helium gas to a continuous jet stream of ∼4 μm in diameter. The X-rays were aligned so as to hit the liquid in the jet region before the Rayleigh breakup of the jet into droplets. All microcrystals were filtered through a 20-μm cutoff filter (VICI AG International) before injection.

Diffraction data were collected at the CXI^16^ beamline at the LCLS in January 2011. Diffraction data were recorded on Cornell-SLAC Pixel Array detectors^9^, consisting of 64 tiles (each 192 pixels by 185 pixels) with a pixel size of 110 × 110 μm^2^. The X-ray wavelength at CXI was 1.32 Å, pulse energy 9.34 keV and pulse duration 42 fs. The XFEL beam was focused to a 10-μm^2^ area spot and the detector distance was 142 mm from the microjet.

### Data processing

Data were processed using the software suite CrystFEL^23^. Data from 1,175 diffraction images were indexed using an orthorhombic unit cell with unit cell axes *a*=57.9 Å, *b*=84.8 Å, *c*=384.3 Å and *α*=*β*=*γ*=90°. As every diffraction image is a still, there are no fully recorded reflections. Scaling and merging of data were thus performed using Monte Carlo methods developed by Kirian *et al.*^24^ In parallel, scaling and merging was also performed by the CCP4 programme Aimless^32^, using the unmerged observations derived by CrystFEL. The same set of unmerged observations was sorted and grouped according to image number or unique hkl indices using the cctbx libraries^33^. Data collection statistics are summarized in Table 1 and Supplementary Table S1, and the multiplicity of 27 is consistent across all shells. When data were split into two equal sets (consisting of even and odd images) and processed in parallel, an *R*_split_ value of 37% (53% outer shell) and an internal correlation coefficient (CC_1/2_) of 0.54 (0.32 outer shell) were recovered when merging the two halves of the data. Errors associated with indexing and integrating data along the long *c* axis contribute to this relatively low value of CC_1/2_ (Supplementary Fig. S1a,b). The value of *R*_split_ is consistent with distributions calculated from subsets of lysozyme data assuming a multiplicity of 27 (Supplementary Fig. S5 of ref. 5).

### Structure determination

For structural analysis, diffraction data were cut at 3.5 Å resolution. PhaserMR 2.5.0 (ref. 34) was used to obtain a molecular replacement solution in the space group P2_1_2_1_2_1_ with a search model based on the high-resolution structure of RC_*vir*_ (PDB entry 2WJN). The best solutions had a translation function *Z* score of 32.8, a rotation function *Z* score of 28.6 and a log likelihood gain of 2,879. The SFX RC_*vir*_ structure was refined using the CCP4 suite. Rigid body refinement was performed using 20 cycles with cofactors omitted, followed by an identical round of refinement but with all cofactors present. Jelly body refinement was used together with several rounds of Translation/Libration/Screw (TLS) refinement, converging to *R*_work_/*R*_free_=0.294/0.327 (figure of merit 0.78). During refinement, we used simple Wilson scaling and a constant density (using default values) was assigned to the region of the unit cell not occupied by protein atoms. We calculated the solvent mask using default parameters: increase van der Waals radius of non-ion atoms by 1.2 Å, increase ionic radius of potential ions by 0.8 Å and shrink the area of the mask by 0.8 Å after the calculation. Crystallographic data and refinement statistics are summarized in Table 1. The geometry was evaluated using the MOLPROBITY^35^ and PROCHECK^36^ yielding 97% in the Ramachandran-favoured region and 3% in the allowed region. mF_obs_−DF_calc_ omit electron density maps (Fig. 4) were calculated by first removing Mg and Fe atoms from the model and then recalculating the map. Composite omit maps were calculated by Phenix^37^, using default parameters (no simulated annealing). All composite omit maps were contoured at 1*σ*.

### Analysis of conformational differences

The refined SFX structure was compared with other crystallographic structures using an error-weighted internal distance matrix analysis^29^, which builds upon the Cruickshank diffraction precision index^38^. For r.m.s.d. comparisons (Supplementary Table S3), RC_*vir*_ structures were first overlaid on their 11 TM helices and the r.m.s.d. calculated on *C*_α_ atoms.

## Author contributions

R.N., L.C.J., H.N.C., J.C.H.S., P.F., I.S. and S.B. conceived the experiment, which was designed by G.J.W., A.B., D.P.D.P., R.B.D, U.W. and R.L.S. Sample preparation and crystallization were performed by L.C.J. and D.A. SFX experiments were carried out by L.C.J., D.A., G.K., T.A.W., A.B., D.P.D.P., R.L.S., C.W., A.S., G.J.W., A.A., M.J.B., C.C., R.B.D., M.F., R.F., L.G., I.G., M.S.H., R.A.K., S.K., C.K., M.L., L.L., E.M., J.D., A.V.M., M.M., K.N., L.R., M.M.S., J.Sj., J.St., F.S., D.W., W.Y.W., U.W., S.W., N.A.Z., S.B., J.C.H.S., I.S., H.N.C., P.F. and R.N. Beamline setup was done by S.B., G.J.W., M.M. and M.M.S. The development and operation of the sample delivery system was performed by R.B.D., D.P.D.P., U.W., R.L.S, L.L. and J.C.H.S. L.C.J., G.K. and A.S. analysed the data. L.C.J. and G.K. performed molecular replacement, refined the structure and calculated the electron density maps. L.C.J. and C.W. analysed the structure. The manuscript was prepared by L.C.J. and R.N. with discussions and improvements from all authors.

## Additional information

**PDB Accession code**: Coordinates and structure factors for the RC_*vir*_ SFX structure have been deposited in the RCSB Protein Data Bank under accession code 4CAS.

**How to cite this article:** Johansson, L. C. *et al.* Structure of a photosynthetic reaction centre determined by serial femtosecond crystallography. *Nat. Commun.* 4:2911 doi: 10.1038/ncomms3911 (2013).

## Supplementary Material

Supplementary Information Supplementary Figures S1-S4, Supplementary Tables S1-S3 and Supplementary References

## Figures and Tables

**Table 1:** Data collection and refinement statistics.

**Data collection and refinement**	**SFX RC**_***vir***_
*Data collection*	
Total number of recorded images	2,744,614
Number of images >10 spots	88,924
Number of confirmed diffraction patterns	5,767
Number of indexed images	1,175
Space group	P2_1_2_1_2_1_
Unit cell parameters	
*a*, *b*, *c* (Å)	57.9, 84.8, 384.3
*α*, *β*, *λ* (°)	90, 90, 90
Resolution (Å)	49.6–2.62
	
*Data collection statistics in the range 50–3.5 Å*	
Completeness (%)	99.1 (93.4)
Multiplicity	27.0 (27.6)
Overall *R*_split_ on *I* (%)	36.5 (52.7)
Mean *I*/*σ*(*I*)	3.50 (2.0)
CC_1/2_*	0.54 (0.32)
	
*Refinement*	
Refinement resolution limits (Å)	49.6–3.50 (3.66–3.50)
Number of unique reflections	24,721
*R*_work_/*R*_free_	29.4/32.7
Overall figure of merit (%)	78
Number of atoms	10,039
Protein	9,224
Ligand/ion	815
Root mean square (bonds, Å)	0.02
Root mean square (angles, °)	1.517
Average temperature factor (Å^2^)	58.51
Wilson B factor (Å^2^)	79.50
Cruickshank diffraction precision index (Å)	0.749

Values in parenthesis refer to the highest-resolution shell (3.66–3.5 Å).

^*^Calculated using Aimless.
