# Supplementary material for: Structure of a photosynthetic reaction centre determined by serial femtosecond crystallography
Source: Nat Commun. 2013 Dec 19;4:2911. doi: 10.1038/ncomms3911 (PMC3905732; doi:10.1038/ncomms3911)
Supplement: Supplementary Information — Supplementary Figures S1-S4, Supplementary Tables S1-S3 and Supplementary References [file ncomms3911-s1.pdf]

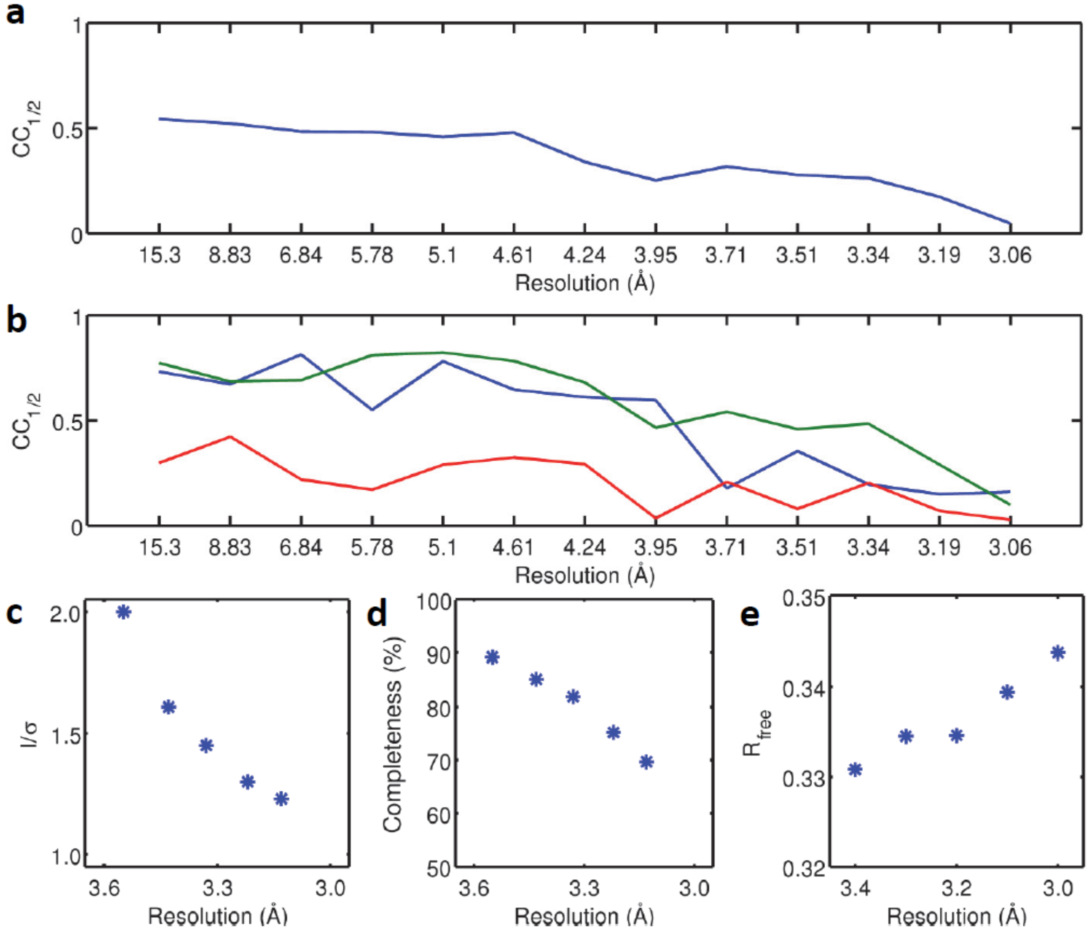

Supplementary Figure S1. Diffraction data quality parameters. (**a**) The overall internal correlation coefficient as a function of resolution calculated using Aimless[32] when data is separated into two sets consisting of only even or only odd images. (**b**) The internal correlation coefficient calculated when data were projected along each of the crystallographic axes. Blue: projection along a*; Green: projection along b*; Red: projection along c*, where a*, b* and c* are the reciprocal lattice vectors. (**c**) I/σ calculated as a function of resolution shell. (**d**) Crystallographic completeness calculated as a function of resolution shell. (**e**) R_{free} values recovered when refining against the SFX crystallographic data cut to the specified resolution.

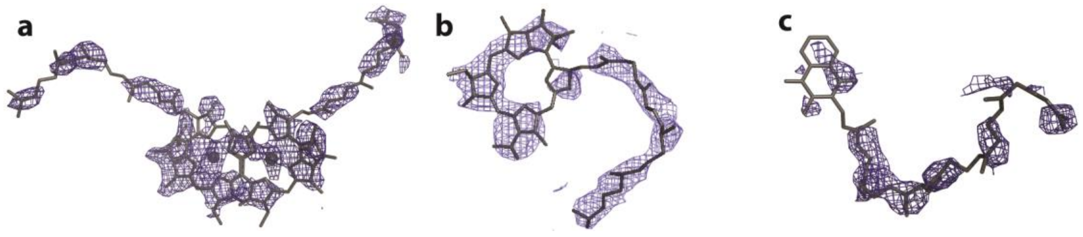

Supplementary Figure S2: Composite omit map calculated at 3.3 Å resolution. Electron density surrounding: (**a**) the special pair; (**b**) the L-branch bacteriopheophytin; and (**c**) the menaquinone. Although the diffraction data were cut to higher resolution than the 3.5 Å resolution composite omit map shown in **Fig. 2e-g**, no additional structural details are apparent and the electron density appears more disjointed. All figures are contoured at 1σ.

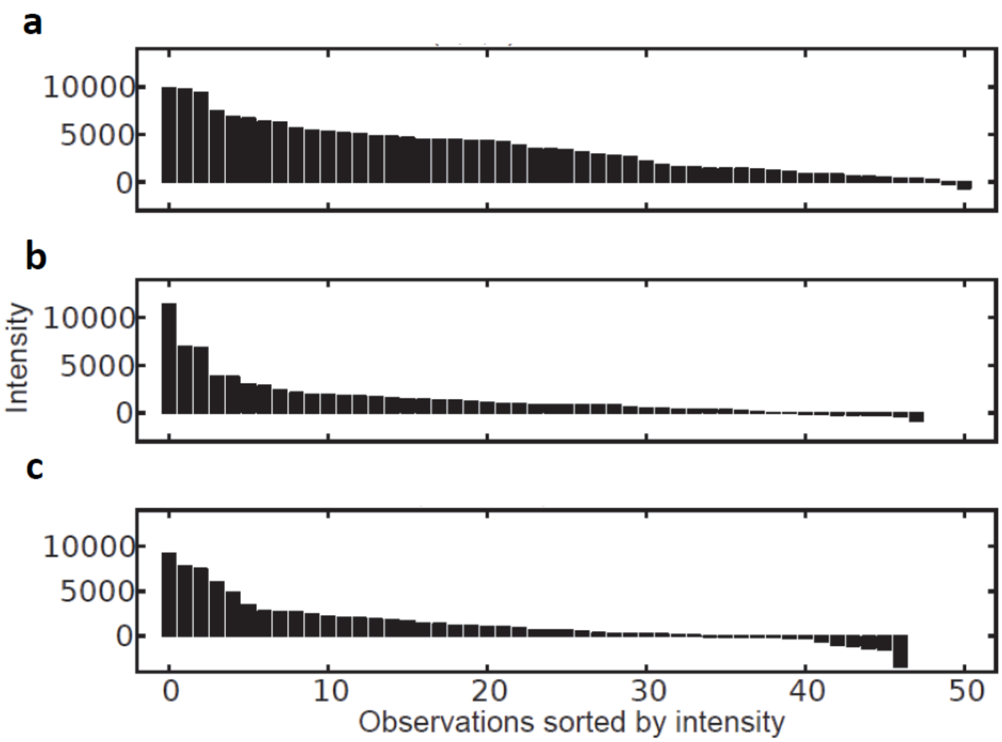

Supplementary Figure S3. Multiple observations of representative reflections recorded from microcrystals of RC_{vir}. These observations were: (**a**) h,k,l = 3,4,5 (14 Å resolution); (**b**) h,k,l = 7,8,9 (6.5 Å resolution); (**c**) h,k,l = 12,13,14 (3.8 Å resolution). For illustrative purposes, these observations are ordered from the strongest positive reflection to the strongest negative reflection. These data were scaled and merged using Monte Carlo methods[24] to recover the experimental SFX intensity $\langle I_{hkl} \rangle$ used for subsequent crystallographic analysis.

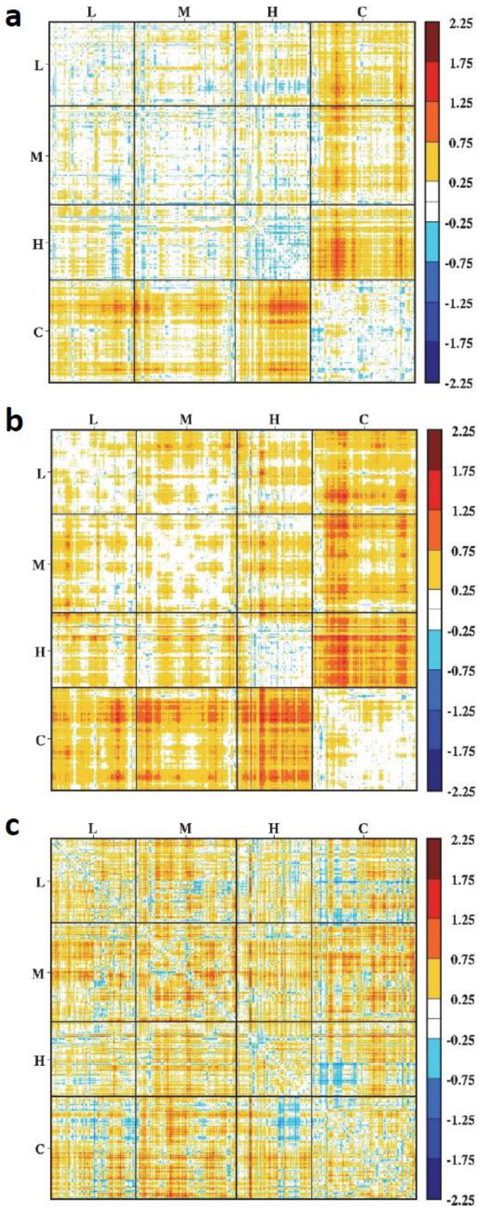

Supplementary Figure S4: Internal distance matrix differences for the RC_{vir} SFX structure versus other LSP crystal structures of RC_{vir} calculated on C_{α} atoms. (**a**) Comparison of the SFX structure with the room-temperature Laue diffraction structure (pdb entries 4CAS - 2X5U). The color code indicates the statistical significance (given in units of σ) of a structural difference calculated after coordinate error weighting.[29] Red implies that the internal distances are larger in

the SFX structure. The strongest differences ($\geq 1.0\ \sigma$) are associated with perturbations of the H- and C-subunits, which participate extensively in crystal contacts (**Fig. 3**). Structural differences within each subunit (the diagonal blocks) are not statistically significant. (**b**) Comparison of the SFX structure with the cryogenic temperature monochromatic data-collection structure (pdb entries 4CAS - 2WJN). (**c**) Comparison of the room temperature Laue diffraction structure with the cryogenic temperature monochromatic data-collection structure (pdb entries 2X5U - 2WJN).

Supplementary Table S1: Crystallographic data statistics by resolution shell

dmax (Å)	No. of unique reflections	Completeness (%)	$R_{split}(I)$ (%)	<I/σ>	Multiplicity
12.31	2629	96.0	26.3	5.2	24.9
6.62	2529	98.2	31.7	4.5	26.7
5.54	2521	99.2	33.5	4.3	28.8
4.94	2459	98.7	33.8	4.1	29.1
4.54	2472	98.1	35.6	4.0	27.4
4.25	2325	95.0	42.0	3.1	26.3
4.02	2377	94.7	46.8	2.7	25.5
3.83	2310	92.9	49.3	2.4	26.6
3.67	2299	94.2	49.4	2.3	27.3
3.54	2315	93.5	52.7	2.0	27.6

Supplementary Table S2: Space group and unit cell axes for other crystal forms of RC_{vir}.

Reference	Resolution (Å)	PDB ID	a	b	C	Space Group
Lancaster *et al.*[39] (2000)	2.0	1DXR	223.5	223.5	113.6	$P\ 4_3\ 2_1\ 2$
Deisenhofer *et al.*[40] (1995)	2.3	1PRC	223.5	223.5	113.6	$P\ 4_3\ 2_1\ 2$
Lancaster *et al.*[41] (2007)	2.4	2JBL	223.5	223.5	113.6	$P\ 4_3\ 2_1\ 2$
Lancaster & Michel.[42] (1997)	2.5	2PRC	223.5	223.5	113.6	$P\ 4_3\ 2_1\ 2$
Lancaster & Michel.[42] (1997)	2.4	3PRC	223.5	223.5	113.6	$P\ 4_3\ 2_1\ 2$
Lancaster & Michel.[43] (1999)	2.4	5PRC	223.5	223.5	113.6	$P\ 4_3\ 2_1\ 2$
Lancaster & Michel.[43] (1999)	2.3	6PRC	223.5	223.5	113.6	$P\ 4_3\ 2_1\ 2$
Lancaster & Michel.[43] (1999)	2.7	7PRC	223.5	223.5	113.6	$P\ 4_3\ 2_1\ 2$
Roszak *et al.*[44] (2012)	1.9	3T6E	221.6	221.6	113.4	$P\ 4_3\ 2_1\ 2$
Roszak *et al.*[44] (2012)	2.0	3T6D	220.4	220.4	113.1	$P\ 4_3\ 2_1\ 2$
Ponomarenko *et al.*[45] (2009)	2.5	3G7F	220.1	220.1	112.7	$P\ 4_3\ 2_1\ 2$
Li *et al.*[46] (2008)	3.2	3D38*	241.2	241.2	113.4	$P\ 3_1\ 2\ 1$
Li *et al.*[47] (2006)	2.0	2I5N	220.4	220.4	113.0	$P\ 4_3\ 2_1\ 2$
Baxter *et al.*[48] (2005)	2.2	1VRN	219.4	219.4	112.6	$P\ 4_3\ 2_1\ 2$
Baxter *et al.*[49] (2004)	2.9	1R2C	223.5	223.5	112.5	$P\ 4_3\ 2_1\ 2$
Wöhri *et al.*[30] (2009)	2.0	2WJM	84.5	138.5	177.8	$P\ 2_1\ 2_1\ 2$
Wöhri *et al.*[30] (2009)	1.9	2WJN	84.8	139.4	178.2	$P\ 2_1\ 2_1\ 2$
Wöhri *et al.*[28] (2010)	3.0	2X5U	85.7	143.5	178.0	$P\ 2_1\ 2_1\ 2$
Wöhri *et al.*[28] (2010)	3.0	2X5V	85.7	143.5	178.0	$P\ 2_1\ 2_1\ 2$
Johansson *et al.*[4] (2012)	8.2	4AC5	57.5	84.6	375.8	$P\ 2_1\ 2_1\ 2_1$
SFX structure (this work)	3.5	4CAS	57.9	84.8	384.3	$P\ 2_1\ 2_1\ 2_1$

* $\gamma = 120°$. All other structures have $\alpha = \beta = \gamma = 90°$.

Supplementary Table S3: Root mean square deviations on C_{α} atoms of the SFX structure (4CAS) relative to other LSP crystal forms.

Subunit(s)	4CAS* – 2X5U¶	4CAS – 2WJN£	2X5U – 2WJN
L+M+H+C	0.49 Å	0.47 Å	0.42 Å
L	0.33 Å	0.36 Å	0.28 Å
M	0.31 Å	0.30 Å	0.28 Å
H	0.48 Å	0.40 Å	0.42 Å
C	0.71 Å	0.68 Å	0.58 Å

*This work
¶Room temperature Laue diffraction.
£Cryo-temperature monochromatic.

SUPPLEMENTARY REFERENCES

39. Lancaster, C.R., Bibikova, M.V., Sabatino, P., Oesterhelt, D. & Michel, H. Structural basis of the drastically increased initial electron transfer rate in the reaction center from a Rhodopseudomonas viridis mutant described at 2.00-A resolution. *J. Biol. Chem.* **275**, 39364-39648 (2000).

40. Deisenhofer, J., Epp, O., Sinning, I. & Michel, H. Crystallographic refinement at 2.3 A resolution and refined model of the photosynthetic reaction centre from Rhodopseudomonas viridis. *J. Mol. Biol.* **246**, 429-457 (1995).

41. Lancaster, C.R., Hunte, C., Kelley, J., 3rd, Trumpower, B.L. & Ditchfield, R. A comparison of stigmatellin conformations, free and bound to the photosynthetic reaction center and the cytochrome bc1 complex. *J. Mol. Biol.* **368**, 197-208 (2007).

42. Lancaster, C.R. & Michel, H. The coupling of light-induced electron transfer and proton uptake as derived from crystal structures of reaction centres from Rhodopseudomonas viridis modified at the binding site of the secondary quinone, QB. *Structure* **5**, 1339-1359 (1997).

43. Lancaster, C.R. & Michel, H. Refined crystal structures of reaction centres from Rhodopseudomonas viridis in complexes with the herbicide atrazine and two chiral atrazine derivatives also lead to a new model of the bound carotenoid. *J. Mol. Biol.* **286**, 883-898 (1999).

44. Roszak, A.W. et al. New insights into the structure of the reaction centre from Blastochloris viridis: evolution in the laboratory. *Biochem. J.* **442**, 27-37 (2012).

45. Ponomarenko, N.S. et al. Structural and spectropotentiometric analysis of Blastochloris viridis heterodimer mutant reaction center. *Biochim. Biophys. Acta* **1788**, 1822-1831 (2009).

46. Li, L. et al. Simple host-guest chemistry to modulate the process of concentration and crystallization of membrane proteins by detergent capture in a microfluidic device. *J. Am. Chem. Soc.* **130**, 14324-14328 (2008).

47. Li, L. et al. Nanoliter microfluidic hybrid method for simultaneous screening and optimization validated with crystallization of membrane proteins. *Proc. Natl Acad. Sci. USA* **103**, 19243-19248 (2006).

48. Baxter, R.H., Seagle, B.L., Ponomarenko, N. & Norris, J.R. Cryogenic structure of the photosynthetic reaction center of Blastochloris viridis in the light and dark. *Acta. Crystallogr. D-Biol. Crystallogr.* **61**, 605-612 (2005).

49. Baxter, R.H. et al. Time-resolved crystallographic studies of light-induced structural changes in the photosynthetic reaction center. *Proc. Natl. Acad. Sci. USA* **101**, 5982-5987 (2004).
